# Assessment of indirect inflammatory markers in patients with myocardial bridging

**DOI:** 10.5830/CVJA-2016-080

**Published:** 2017

**Authors:** Levent Cerit

**Affiliations:** Department of Cardiology, Near East University, Nicosia, Cyprus

**Keywords:** myocardial bridging, platelet distribution width, neutrophil-to-lymphocyte ratio

## Abstract

**Introduction:**

Myocardial bridging (MB) is a congenital variant of the coronary artery in which a portion of the epicardial coronary artery takes an intramuscular course. Although it is considered a benign anomaly, it may lead to such complications as myocardial ischaemia, acute coronary syndrome, coronary spasm, exercise-induced dysrhythmias or even sudden death. MB may be related to increased inflammatory and atherosclerotic processes. This study was conducted with the aim of evaluating the relationship between neutrophil/ lymphocyte ratio (NLR) and MB.

**Methods:**

Taking into consideration the inclusion criteria, 86 patients with MB and 88 with normal coronary angiographies (control group) were included in the study. The association between MB and laboratory and other clinical parameters was evaluated.

**Results:**

The platelet distribution width (PDW) (17.3 ± 0.40 vs 16.1 ± 0 .5; p < 0.05), NLR (3.2 ± 1.3 vs 2.2 ± 0.9; p < 0.05) and red cell distribution width (RDW) (14.3 ± 1.3 vs 13.1 ± 1.1; p < 0.05) were significantly higher in the MB group than in the control group.

**Conclusions:**

This study demonstrated that compared to normal coronary arteries, PDW, NLR and RDW were significantly higher in MB patients. Further studies are needed to clarify the increased inflammatory parameters in patients with MB.

## Introduction

Myocardial bridging (MB) is an anatomical variation characterised by narrowing during systole of some of the epicardial coronary arterial segments running in the myocardium. It may be encountered in 0.5 to 16% of routine coronary angiographies.^1^-^3^ Although it is considered a benign anomaly, it may lead to such complications as myocardial ischaemia, acute coronary syndromes, coronary spasm and exercise-induced dysrhythmias, such as supraventricular tachycardia, ventricular tachycardia, syncope or even sudden death.^4^,^5^

Platelet distribution width (PDW) is a direct measure of the variation in platelet size and a marker of platelet activation.^6^ Red cell distribution width (RDW) is a direct measure of the variation in erythrocyte size, which is measured as a component of routine blood counts.^7^ The RDW is a well-recognised indicator of chronic inflammation and oxidative stress, and elevated RDW is strongly associated with poor clinical outcomes among patients with coronary artery disease (CAD).^8^ The neutrophil/lymphocyte ratio (NLR), derived from the white blood cell (WBC) count, is a common prognostic indicator in cardiovascular disease.^9^

The aim of this study was to evaluate the relationships between MB and PDW and other haematological parameters in an effort to identify useful clinical indicators in patients undergoing coronary angiography.

## Methods

A retrospective evaluation was conducted of consecutive patients undergoing coronary angiography. Stable angina was defined as discomfort in the chest, back, shoulder, jaw or arms, typically elicited by exertion or emotional stress, and relieved by rest or nitroglycerin.

All patients enrolled in the study underwent coronary angiography as a result of chest pain and objective signs of ischaemia during treadmill exercises. Routine laboratory and clinical parameters (e.g. hypertension, hypercholesterolaemia, diabetes mellitus, tobacco use, family history of cardiovascular disease) were obtained from the patients’ medical records.

Study exclusion criteria included CAD, mild-to-severe valve disease, heart failure, anaemia, renal failure, inflammatory diseases, coronary ectasia, malignancy, peripheral and cerebral arterial disease and thyroid gland dysfunction (hypohyperthyroidism).

All patients underwent transthoracic echocardiography using the Vivid S5 (GE Healthcare) echocardiography device and Mass S5 probe (2–4 MHz). Standard two-dimensional and colour-flow Doppler views were acquired according to the guidelines of the American Society of Echocardiography and European Society of Echocardiography.^10^ The ejection fraction was measured according to the Simpson’s method.^10^

Coronary angiography was performed with the Judkins technique11 and Innova 3100-IQ angiographic system (General Electric, Buc Cedex, France). A typical description of bridging on angiographyic view involves systolic narrowing, or ‘milking’ of an epicardial artery, with a ‘step-down’ and ‘step-up’ demarcating the impacted area. Angiographic views were evaluated based on these MB criteria, and ≥ 50% systolic narrowing of an epicardial artery was considered MB. Coronary angiograms were assessed independently for objective evaluation of MB by two invasive cardiologists blinded to the clinical findings.

Prior to coronary angiography, eight-hour postprandial venous blood was collected from all patients for routine laboratory testing. Complete blood counts (CBC), including haemoglobin, haematocrit and WBC count were analysed using an automated CBC device (Abbott Cell Dyn; Abbott Laboratories, Effingham, Illinois, USA). Biochemical parameters were measured using an Olympus AU 600 auto-analyzer (Olympus Optical Co. Ltd, Schimatsu-Mishima, Japan). All study parameters were reviewed and approved by the local ethics committee.

## Statistical analysis

Statistical analysis was performed using the SPSS (version 20.0, SPSS Inc, Chicago, Illinois) software package. Continuous variables are expressed as mean ± standard deviation (mean ± SD) and categorical variables as percentage (%). The Kolmogorov– Smirnov test was used to evaluate the distribution of variables. The Student’s t-test was used to evaluate continuous variables showing normal distribution and the Mann–Whitney U-test to evaluate variables that did not show normal distribution. A p-value < 0.05 was considered statistically significant

## Results

The study population consisted of 1 368 consecutive patients undergoing coronary angiography. Out of the total population, 86 patients with MB were included in the study group. The control group consisted of 88 age-matched subjects with normal coronary angiograms, selected consecutively during the same study period as the study group. The same exclusion criteria were applied to the study and control groups.

The distribution of cardiovascular risk factors, demographic characteristics, and laboratory parameters in the two groups are shown in Table 1. The mean age of the MB group was 56 ± 9 years and control group was 54 ± 7 years (p = 0.468).

**Table 1:** Distribution of baseline characteristic of all patients

*Variables*	*Normal coronary artery (n = 88)*	*Myocardial bridging (n = 86)*	*p-value*
Age (years)	54 ± 7	56 ± 9	0.468
Male gender, n (%)	58 (66)	62 (72)	0.342
Family history, n (%)	28 (32)	24 (28)	0.580
Hyperlipidaemia, n (%)	19 (22)	22 (25)	0.385
Smoking, n (%)	23 (26)	21 (24)	0.486
Diabetes mellitus, n (%)	16 (18)	19 (22)	0.385
Hypertension, n (%)	22 (25)	31 (36)	0.034
SBP (mmHg)	121 ± 11	125 ± 8	0,548
DBP (mmHg)	78 ± 9	81 ±6	0.783
Heart rate (bpm)	74 ± 15	78 ±9	0.673
Ejection fraction (%)	62.4 ± 3.1	60.2 ± 4.2	0.471

Values are mean (± SD), SBP: systolic blood pressure, DBP: diastolic blood pressure.

There was no statistically significant difference between the two groups with regard to known CAD risk factors, such as diabetes mellitus and smoking history, except hypertension was more prevalent in the MB group than in the control group (25 vs 36%, p = 0.034; Table 1). The ejection fraction was similar between the two groups (62.4 ± 3.1 vs 60.2 ± 4.2%, p = 0.471; Table 1). The PDW (17.3 ± 0.4 vs 16.1 ± 0.5%, p = 0.003), NLR (3.2 ± 1.3 vs 2.2 ± 0.9%, p = 0.034), and RDW (14.3 ± 1.3 vs 13.1 ± 1.1%, p = 0.032) were significantly increased in the MB group relative to the control group (Table 2).

**Table 2:** Distribution of the haematological parameters of all cases

*Variables*	*Normal coronary artery (n = 88)*	*Myocardial bridging (n = 86)*	*p-value*
White blood cells (103/ μl)	7.9 ± 2.1	8.1 ± 2.3	0.278
Mean corpuscular volume (fl)	88.9 ± 8.3	86.9 ±7.8	0.878
Platelets (× 1 000/mm^3^)	266 ± 38	272 ± 41	0.647
Haemoglobin (g/dl)	13.8 ± 1.9	14.1 ± 1.3	0.387
RDW (%)	13.1 ± 1.1	14.3 ± 1.3	0.032
Mean platelet volume (fl)	8.8 ± 0.9	8.9 ± 1.1	0.093
Platelet distribution width (%)	16.1 ± 0.9	17.3 ± 1.1	0.003
NLR	2.2 ± 0.9	3.2 ± 1.1	0.034

RDW: red blood cell distribution width, NLR: neutrophil-to-lymphocyte ratio.

## Discussion

In this study we examined the relationship between MB and PDW and other haematological parameters. MB was independently associated with increased values of PDW, NLR and RDW.

MB is a congenital variant of the coronary artery in which a portion of the epicardial coronary artery takes an intramuscular course.^12^ This arrangement of a ‘tunnelled’ segment of the artery under the ‘bridge’ of overlying myocardium frequently results in vessel compression during systole. While this condition is frequently asymptomatic, in many cases it may be responsible for adverse complications, including coronary atherosclerosis, angina, myocardial ischaemia,^13^ acute coronary syndromes,^14^-^16^ left ventricular dysfunction and stunning,^17^ arrhythmias,^18^ and even sudden cardiac death.^19^

Early pathological analysis of myocardial bridging recognised ‘sparing’ of the bridged segments from atherosclerotic lesions.^20^ The intima of the tunnelled segment is significantly thinner than the proximal segment, and includes a predominance of the ‘contractile’ subtype of smooth muscle cells, thought to be negatively associated with progression of atherosclerotic lesions.^21^ In addition, known as vasoactive agents, endothelial nitric oxide synthase, endothelin-1 and angiotensin-converting enzyme levels are decreased in the bridged coronary wall.^22^ These agents have been implicated in the proliferation of smooth muscle cells, resulting in increased size of atherosclerotic lesions. Systolic kinking of the bridged segments and endothelial dysfunction may also predispose to coronary vasospasm and thrombus formation.^23^

Conversely, the proximal segment of the bridge appears to develop atherosclerosis at an increased rate, approximately 90%.24 Endothelial cell morphology at the entrance to the tunnelled segment reveals a ‘flat, polygonal and polymorphic’ structure, indicative of a low-shear stress state, while the endothelial cells within the tunnel maintain a helical orientation, a sign of laminar flow and high shear.^24^ This suggests a haemodynamic basis for the increased plaque formation proximal to the tunnel, through impairment of endothelial cell function and morphology. Also, expression of the vasoactive agents, endothelial nitric oxide synthase, endothelin-1 and angiotensin-converting enzyme are Table 1. Distribution of baseline characteristic of all patients all increased in the proximal segment.^22^

PDW is a more specific indicator of platelet activation than mean platelet volume in the absence of platelet swelling.^25^ Elevated PDW directly measures the variability in platelet size during platelet distension and serves as a marker of platelet activation.^26^ Increased platelet number and size, and the presence of pseudopodia may influence PDW. Significant elevation of PDW has been observed among patients with acute myocardial infarction and unstable angina pectoris.^27^

Jindal et al.^28^ reported a significant association between PDW and microvascular dysfunction among diabetic patients. Numerous factors contribute to microvascular and circulatory dysfunction, including coronary microvascular imbalance and increased tonus, endothelial thickening of small vessels and endothelial nitric oxide imbalance, and blood viscosity.

In our study, we detected higher PDW levels in patients with MB. We assumed that a higher PDW level was related to increased vasoactive agents, including endothelial nitric oxide synthase, endothelin-1 and angiotensin-converting enzyme in the proximal segment of the MB.

The NLR is related to the development of atherosclerosis in the coronary arteries,^29^ and NLR is an excellent indicator of cardiovascular disease.^9^ Among patients with acute coronary syndrome, neutrophils are functionally activated, and the presence of localised neutrophil infiltration in atherosclerotic lesions has been demonstrated, assuming that neutrophils play a key role in the mediation and destabilisation of atherosclerotic plaques.^30^ Our study demonstrated a significant correlation between the presence of MB and NLR, an inflammatory marker linked to early atherosclerosis.

Chronic inflammation may act synergistically to raise RDW and augment the atherosclerotic process.^31^ The RDW is an independent predictor of mortality and coronary morbidity among patients with myocardial infarction.^8^ In our study, higher RDW was found in patients with MB than in the control group.

The relationship between cardiovascular disease and increased platelet activity is well known. In this study, we found a significant relationship between MB and PDW, an established indicator of platelet activity. Additionally, we found a significant relationship between MB and NLR, an indicator of systemic inflammation. These predictive parameters are easily measured and are inexpensive in routine clinical practice.

There were some limitations to this study. We evaluated the coronary arteries using coronary angiography. Although it is well known that intravascular ultrasound (IVUS) provides a more accurate evaluation of coronary atherosclerosis, we were unable to perform IVUS assessments.

Coronary atherosclerosis is present anatomically in approximately 25% of patients, based on autopsy and computed tomography (CT), but results in angiographically detectable systolic compression in less than 10% of patients. Cardiac CT angiography may be a useful tool to more precisely detect MB, however, we were unable to perform cardiac CT angiography in this study. In addition, the study data are reflective of the crosssectional design and may not reflect the long-term clinical status of the patients.

## Conclusion

To the best of our knowledge, this study is the first to evaluate the relationship between MB and indirect inflammatory markers. Our study reveals a significant association between indirect inflammatory markers and MB. Further studies are needed to clarify the relationship between MB and indirect inflamatory markers.
